# The association of hypertriglyceridemia with cardiovascular events and pancreatitis: a systematic review and meta-analysis

**DOI:** 10.1186/1472-6823-12-2

**Published:** 2012-03-31

**Authors:** M Hassan Murad, Ahmad Hazem, Fernando Coto-Yglesias, Svitlana Dzyubak, Shabnum Gupta, Irina Bancos, Melanie A Lane, Patricia J Erwin, Lars Berglund, Tarig Elraiyah, Victor M Montori

**Affiliations:** 1 Knowledge and Evaluation Research Unit, Mayo Clinic, Rochester, MN, USA; 2 Division of Preventive Medicine, Mayo Clinic, Rochester, MN, USA; 3 Department of Internal Medicine, University of North Dakota, Fargo, ND, USA; 4 Davis and the VA Northern California Health Care System, University of California, Sacramento, USA; 5 Division of Endocrinology, Diabetes, Metabolism, Nutrition, Mayo Clinic, Rochester, MN, USA

**Keywords:** Hypertriglyceridemia, Cardiovascular disease, Pancreatitis, Systematic reviews and meta-analysis

## Abstract

**Background:**

Hypertriglyceridemia may be associated with important complications. The aim of this study is to estimate the magnitude of association and quality of supporting evidence linking hypertriglyceridemia to cardiovascular events and pancreatitis.

**Methods:**

We conducted a systematic review of multiple electronic bibliographic databases and subsequent meta-analysis using a random effects model. Studies eligible for this review followed patients longitudinally and evaluated quantitatively the association of fasting hypertriglyceridemia with the outcomes of interest. Reviewers working independently and in duplicate reviewed studies and extracted data.

**Results:**

35 studies provided data sufficient for meta-analysis. The quality of these observational studies was moderate to low with fair level of multivariable adjustments and adequate exposure and outcome ascertainment. Fasting hypertriglyceridemia was significantly associated with cardiovascular death (odds ratios (OR) 1.80; 95% confidence interval (CI) 1.31-2.49), cardiovascular events (OR, 1.37; 95% CI, 1.23-1.53), myocardial infarction (OR, 1.31; 95% CI, 1.15-1.49), and pancreatitis (OR, 3.96; 95% CI, 1.27-12.34, in one study only). The association with all-cause mortality was not statistically significant.

**Conclusions:**

The current evidence suggests that fasting hypertriglyceridemia is associated with increased risk of cardiovascular death, MI, cardiovascular events, and possibly acute pancreatitis.

Précis: hypertriglyceridemia is associated with increased risk of cardiovascular death, MI, cardiovascular events, and possibly acute pancreatitis

## Background

Hypertriglyceridemia is a manifestation of several common metabolic disorders in the western world. A recent cross-sectional study found that over 33% of adults in the United States had hypertriglyceridemia (serum triglyceride levels over 150 mg/dl (1.7 mmol/L)) of whom over 50% had serum triglyceride levels exceeding 200 mg/dl (2.2 mmol/L) [1].

The association of hypertriglyceridemia and clinically important complications such as cardiovascular events and acute pancreatitis has been suggested by several studies. Previous epidemiologic studies demonstrated increase in the risk of cardiovascular events although there has always been significant confounding due to varying levels of adjustments for traditional risk factors and other lipid subfractions [2-4]. As for pancreatitis, case series and uncontrolled studies reported that very severely elevated triglyceride levels are associated with lipemic serum, chylomicronemia syndrome, and increased risk of pancreatitis [5-7]. Serum triglycerides levels of 1000 mg/dl (11.3 mmol/L) and higher have been observed in 12% to 38% of patients presenting with acute pancreatitis [5]. However, the association with pancreatitis has not been evaluated in controlled studies or with less severe hypertriglyceridemia.

To update the evidence base to the present time (last meta-analysis [2] was performed 6 years ago), we conducted this systematic review and meta-analysis. Our goal was to assess the magnitude of association and the quality of supporting evidence linking hypertriglyceridemia with cardiovascular events, mortality and pancreatitis. We specifically aimed at comparing association measures in studies with varying levels of adjustment for cardiovascular risk factors and to search for controlled studies evaluating the risk of pancreatitis.

## Methods

This systematic review was conducted according to a priori established protocol that was commissioned and funded by the Endocrine Society and is reported according to the PRISMA statement (Preferred Reporting Items for Systematic Reviews and Meta-analyses) [8].

### Eligibility criteria

Eligible studies were randomized and observational studies that enrolled patients with untreated hypertriglyceridemia and reported a relative association measure between fasting serum triglycerides levels and the outcomes of interest: all-cause mortality, cardiovascular death, cardiovascular events and pancreatitis. We excluded uncontrolled studies and studies of nonfasting hypertriglyceridemia.

### Study identification and data extraction

An expert reference librarian (P.J.E) created and implemented the electronic search strategy with input from study investigators (V.M.M. & M.H.M). We searched Ovid MEDLINE, Ovid EMBASE, Web of Science and SCOPUS through August of 2010. The detailed search strategy is available in Additional file 1. We also sought recommendations from content expert for potentially relevant studies to be included in the screening process.

Reviewers working independently and in duplicate assessed each abstract for eligibility. Disagreements yielded an automatic inclusion into the following level of screening. Included studied were retrieved and full text screening commenced in duplicate as well. Disagreements in this level were resolved by discussion and consensus. Online reference management system was used to conduct this review and it reported a real-time chance-adjusted agreement (kappa) statistic to evaluate the agreement among reviewers. Kappa averaged 0.80. Two reviewers working independently and in duplicate extracted baseline and outcome data and assessed the quality of included study. A third reviewer compared the reviewer's data and resolved inconsistencies by referring to the full text article.

### Quality

Using the Newcastle-Ottawa scale, [9] reviewers assessed the quality of included observational studies (and control arms of RCT, considered as observational cohorts) by determining outcome ascertainment, adjustment for confounders, proportion of patients lost to follow-up as well as sample selection. We used the GRADE approach in evaluating the evidence yielded from included studies[10].

### Statistical analysis

We pooled the relative association measures of relevant complications from included studies and analyzed the data using the random-effects model described by DerSimonian and Laird [11]. Heterogeneity in results across studies was measured using the *I^2 ^*statistic, which estimates the proportion of variation in results across studies that is not due to chance. An *I^2 ^*of 50% or more indicates large inconsistency between studies. Meta-analysis was completed using Comprehensive Meta-analysis (CMA) version 2.2 (Biostat Inc., Englewood, NJ).

#### Subgroup analyses and publication bias

*A priori *hypotheses were designed to explain between-study inconsistencies in results. These analyses sought an interaction with whether triglycerides levels were adjusted for other lipid fractions or not; whether the underlying metabolic disorder was diabetes vs. not; and whether the association differed between men and women. Publication bias was evaluated by assessing the symmetry of funnel plots and using Egger's regression test. In this regression, the size of the treatment effect is captured by the slope of the regression line and bias is captured by the intercept [12].

## Results

### Search results and included studies

**Figure 1:**
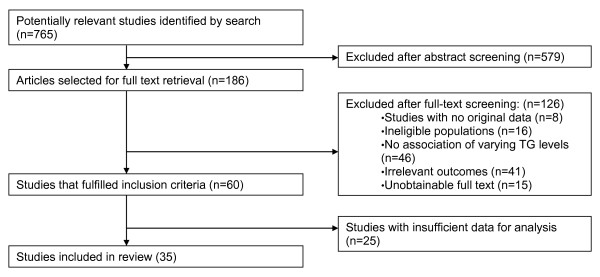
**Study selection process**.

Electronic search yielded 760 potentially eligible studies. Following screening, 60 studies met inclusion criteria, of which 35 reported data sufficient for meta-analysis [Figure 1].

#### Methodological quality and risk of bias

Included studies had a fair methodological quality (Table 2) with follow-up period reported by 85% of studies averaging 114 months; 58% of studies reported loss to follow-up of participants that ranged 0% to 27%. Adjustment for potential confounders was reported in 90% of studies and the outcome ascertainment method was reported in all studies. Cohort selection was random in 18% of the studies.

**Table 1:** Baseline Characteristics of Included Studies

Study Label	Design	Objective of Study	Population	Age (mean)	Sample Size	Length of Follow-up	Definition of CV events
Acarturk, 2004[13]	Prospective cohort	to investigate the relation between age and gender differences in plasma TG and CAD in patients with angiographically proven CAD	patients admitted for diagnostic coronary angiography due to chest pain	54.9 +/-10.26	937	NR	Coronary artery disease

Bansal, 2007[14]	Prospective cohort	To determine the association of triglyceride levels (fasting vs nonfasting) and risk of future cardiovascular events.	healthy women	54.2 +/- 7.06	26,509	136.8 Months (median)	composite of confirmed nonfatal MI, nonfatal ischemic stroke, coronary revascularization, or death due to cardiovascular causes

Barrett-Connor, 1987[15]	Prospective cohort	To examine the independent effect of triglyceride on the prediction of cardiovascular disease after the effects of cholesterol and other heart disease risk factors have been accounted for	healthy fasting men without known CVD	57.7	1,589	144 months	N/A

Bass, 1993[16]	Prospective cohort	To further explore the relationships between lipid and lipoprotein levels and other conventional CVD risk factors and CVD death on women	women 30 years of age and older	58.2 +/- 5.5	1,405	Mean 168 months	N/A

Bonaventure, 2010[17]	Prospective cohort	To find the association pattern between serum TG and incident intracerebral hemorrhage as compared with coronary events and ischemic stroke	Population- based, elderly participants free from institutionalization were recruited from the electoral rolls of three French cities	74.03 years	8,393	mean of 5 years	MI, hospitalized angina pectoris, acute coronary syndrome, coronary endovascular dilatation, coronary bypass, or death due to a coronary event

Carlson, 1988[18]	RCT	To obtain a pronounced serum lipid lowering by combined use of clofibrate and nicotinic acid in an effort to reduce the risk of IHD	Survivors of MI < 70 years of age	58.9 + -0.4 males and 62.5 + -0.9 females	Control group (n = 276)	60 months	N/A

Chan, 2005[19]	Prospective cohort	To examine the lipid profiles in Chinese type 2 diabetic patients and their relationship with anthropometric parameters, glycemic control and cardiovascular mortality.	Chinese patients with type 2 DM	54.0 +/- 14.0	517	Mean 55.2 +/-10.8 months	N/A

Chester, 1995[20]	Prospective cohort	To determine the standard clinical or angiographic variables or both present at initial angiography associated with the development of adverse coronary events in patients awaiting routine PTCA	Patients awaiting routine percutaneous transluminal coronary angioplasty (PTCA)	57	215	Median 8 months	fatal or non-fatal MI, unstable angina or angiographic new total coronary occlusion

Czernichow, 2007[21]	Prospective cohort	To investigated the relationship of baseline 'hypertriglyceridemic waist' (HTGW) status with CVD risk in middle-aged French men	middle-aged French men, included diabetics	51.9 +/- 4.7	3,430	90 months	new-onset angina, fatal and non-fatal MI or stroke, transient ischemic attack, sudden death or intermittent claudication

Drexel, 2005[22]	Prospective cohort	To evaluate the atherogenicity of lipids in coronary patients with normal fasting glucose (NFG), impaired fasting glucose (IFG), and type 2 DM	Caucasian patients who were referred to coronary angiography	62.4 +/- 10.6	750	27.6 +/- 4.8 months	N/A

Eberly, 2003[23]	Prospective cohort	To determine whether HTG is an independent risk factor for coronary heart disease (CHD), and whether fasting and nonfasting triglyceride (TG) levels are equally predictive	men at increased risk but without clinical evidence of definite CHD at baseline	46.3	2809	304.8 months	either a clinical MI or a significant serial electrocardiogram change indicative of MI

Egger, 1999[24]	Prospective cohort	To assess the influence of differential precision in the measurement of the correlated variables total cholesterol and HDL cholesterol on estimates of risk of IHD associated with TG levels	Middle aged men living in the town of Caerphilly, South Wales, UK	52.1 +/- 4.48	2,512	5 and 10 years after baseline	death from ischemic heart disease, clinical non-fatal MI, electrocardiographic MI

Ellingsen, 2003[25]	Prospective cohort	to examine the effect of group assignment on IHD mortality in subjects with normal or high fasting TG	healthy men who had an elevated serum total cholesterol concentration or coronary risk score	46 +/-3	1232	276 months	N/A

Gaziano, 1997[26]	Case controlled study	To examine the interrelationships of the fasting TG level other lipid parameters and nonlipid risk factors with risk of MI.	Patients - coronary care and other intensive care units patients (no history of MI and angina pectoris) with whom symptoms of MI had begun 24 h of admission, control - residents of home towns.	57.7 +/- 9.65	680	NR	N/A

Goldberg, 2009[27]	Prospective/ case controlled	To ascertain coronary artery disease outcomes and predictive factors in patients with SLE and matched healthy controls prospectively	Patients with systemic lupus erythematosus (SLE) and matched healthy controls	SLE cases 44.2 +/-12.2, controls 44.5 +/- 4.4	237 controls and 241 SLE cases	86.4 months	Defined as the occurrence of MI and/or angina pectoris due to atherosclerosis.

Habib, 2006[28]	Prospective cohort	To evaluate the association of serum TC and TG with clinical outcomes in chronic peritoneal dialysis (PD) patients.	Patients on chronic peritoneal dialysis; only in end-stage renal disease (ESRD) or patients those very ill patients who died rapidly due to unrelated conditions.	57.2 +/ 15.3	1,053	23 +/- 14 months	N/A

Haim, 1999[29]	Prospective cohort	To investigate the association between elevated blood triglyceride levels and subsequent mortality risk in patients with established coronary heart disease (CHD)	patients with a diagnosis of CHD	59.76 +/- 6.96	11,546	61.2 months	N/A

Hoogeveen, 2001[30]	Case controlled study	To determine the effect of immigration to the USA ion plasma levels of lipoprotein a and other independent risk factors for CHD in Asian Indians	Asian Indians and Asian Indians living in the USA with and without CHD	44.2 +/- 12.79	309	NR	Coronary heart disease - incidents not specifically defined

Jonsdottir, 2002[31]	Prospective cohort	To examine the relationship between the relative risk of baseline variables and verified MI or coronary death in individuals with no prior history of MI	male and female from Reykjavik and adjusted communities	52.7 +/- 8.71	18,569	Mean 208.8 months	N/A

Lamarche, 1995[32]	Prospective cohort	To confirm the importance of both elevated plasma cholesterol and decreased high density lipoprotein cholesterol levels as risk factors for ischemic heart disease	men without ischemic heart disease	57.5	2,103	60 months	Effort angina pectoris, coronary insufficiency, nonfatal MI, and coronary death

Lloret Linares, 2008[33]	Retrospective cohort	to assess retrospectively the prevalence and the predictive factors of acute pancreatitis (AP)	Patients referred by their general practitioner or general hospital for very high TG levels.	47 +/- 10.7	129	NR	N/A

Lu, 2003[34]	Prospective cohort	To determine whether non- HDL cholesterol, a measure of total cholesterol minus HDL cholesterol, is a predictor of CVD in patients with DM	American Indians with DM	57.28 +/- 8	2,108	108 months	Coronary heart disease, MI, stroke, and other CVD

Malone, 2009[35]	Prospective cohort	This study evaluated cardiometabolic risk factors and their relationship to prevalent diagnosis of acute MI (AMI) and stroke.	People continuously receiving health insurance benefits during study	56.8 +/- 0.03	170,648	24 months	N/A

Mazza, 2005[36]	Prospective cohort	To evaluate whether TG level is a risk factor for CHD in elderly people from general population, and to look for interactions between TG and other risk factors.	elderly people from general population CHD in elderly people from general population	73.8 +/- 5	3,257	144 months	N/A

Mora, 2008[37]	Prospective cohort	To evaluate levels of lipids and apolipoproteins after a typical meal and to determine whether fasting compared with non-fasting alters the association of these lipids and apolipoproteins with incident CVD.	Healthy women, aged > = 45 years, who were free of self- reported CVD or cancer at study entry and with follow-up for incident CVD.	54.7	26,330	136.8 months	Nonfatal MI, percutaneous coronary intervention, coronary artery bypass grafting, nonfatal stroke, or cardiovascular death

Noda, 2010[38]	Case controlled study	To examine the prediction of coronary risk factors and evaluation of the predictive value for MI among Japanese middle-aged male workers.	Japanese male workers	cases 50.4 + -5.3, controls 50.4 + -5.5 years	cases 204 and controls 408	36 months	N/A

Rubins, 1999[39]	RCT	To analyze the role of raising HDL cholesterol level and lowering triglyceride levels to reduce the rate of coronary events in patients with existing cardiovascular disease	men with coronary heart disease with absence of serious coexisting conditions	64 + -7	1267 (placebo)	61.2 months	combined incidence of nonfatal MI or death from coronary heart disease

Samuelsson, 1994[40]	Prospective cohort	To analyze the importance of DM and HTG as potential risk factors for CHD in middle-aged, treated hypertensive men	middle aged treated hypertensive men	52 +/- 2.3	686	180 months	Non-fatal MI, a fatal MI, a death certificate statement of coronary atherosclerosis as the cause of death

Schupf, 2005[41]	Prospective cohort	To investigate the relationship between plasma lipids and risk of death from all causes in non demented elderly	Community- based sample of Medicare recipients without dementia	76.1	2,277	Mean 36 +/- 30 months	N/A

Sprecher, 2000[42]	Prospective cohort	To evaluate the predictive value of serum triglyceride levels on mortality in post coronary artery bypass graft(CABG) diabetic patients with subsequent analysis by sex	Diabetic post CABG patients at a large metropolitan hospital	63 +/- 9	1,172	84 months	N/A

Tanko, 2005[43]	Prospective cohort	To investigate the relative utility of enlarged waist combined with elevated TG (EWET) compared with the National Cholesterol Education Program (MS-NCEP) criteria in estimating future risk of all-cause and cardiovascular mortality	Postmenopausal women	60.4 +/- 7.1	557	8.5 +/- 0.3 years	N/A

Tsai, 2008[44]	Retrospective cohort	To assess the effect of a single and a combination of "pre-disease" risk factors of metabolic syndrome on the overall and cardiac mortality.	civil servants and teachers 40 years and older	52.4 + - 8.0	35,259	median follow-up of 15 years	N/A

Upmeier, 2009[45]	Prospective cohort	To determine whether high levels of serum total cholesterol and low levels of HDL are related to increased mortality in elderly	Home dwelling older adults residents in Finland	70 years	877	144 months	N/A

Valdivielso, 2009[46]	Prospective cohort	To study the prevalence, risk factors and vascular disease associated with moderate and sever HTG in an active working population	Active working population of Spain	36 ± 10 years	594,701	NR	documented prior medical diagnosis of heart disease, cerebrovascular disease or peripheral arterial disease

Wier, 2003[47]	Prospective cohort	To investigate the relationship between triglyceride and stroke outcome	nondiabetic patients presenting to acute stroke unit	Median 70 years	1310	mean 1195 days	N/A

UC/NR: unclear, not reported; TG: Triglycerides; HTG, hypertriglyceridemia; MI, myocardial infarction; DM, diabetes, BP, blood pressure, HTN, hypertension

Triglycerides Conversion: from mg/dL to mmol/L: multiply by (x) 0.01129; from mmol/L to mg/dL: multiply by (x) 88.6

**Table 2:** Quality of Included Studies

Study Label	Cohort Selection (sampling)	Outcome ascertainment*	Adjustments for variables	% lost to follow-up	Definition of hypertriglyceridemia
Acarturk, 2004[13]	not random; all patients admitted for diagnostic coronary angiography	chart review, angiography results	NR	NR	TG value in the blood was used as a continuous number (variable). OR expresses increased risk per unit of serum TG level

Bansal, 2007[14]	derived from women health study, previously completed randomized controlled trial of aspirin and vitamin E	chart review, events adjudicated by an end point committee	adjusted for treatment assignment to ASA, vitamin E, beta carotene, age, BP, smoking status, and use of hormone therapy, levels of total cholesterol and HDL-C, history of DM, BMI, high-sensitivity C- reactive protein	0	TG value in the blood was used as a continuous number (variable). OR expresses increased risk per unit of serum TG level

Barrett-Connor, 1987[15]	random sample	chart review, ICD or billing codes, death certificates	adjust by TG level, age, BP, BMI, smoking habit, DM, family history of heart attack	0.5%	Compared normal to "borderline HTG", defined as TG between 240-500 mg/dL (2.7-5.65 mmol/L)

Bass, 1993[16]	subset of female participants in the Lipid Research Clinics' Follow-up Study	chart review, annual checkups	Adjusted for age, HTN, DM, smoking, history of heart disease and estrogen use	NR	Compared TG < 200 mg/dL (< 2.25 mmol/L) to elevated 200 to 399 mg/dL (2.25 to 4.49 mmol/L) and high > 400 mg/dL (> 4.50 mmol/L)

Bonaventure, 2010[17]	not random and not consecutive: recruited from electoral rolls	Death certificates and autopsy reports, ascertained the same way in cases and controls	medical history of MI, stroke, or peripheral arterial disease, as well as smoking and alcohol consumption status (never, former, current), excess weight, elevated BP, DM, apolipoprotein E (APOE) genotype, low-dose aspirin intake, and lipid-lowering treatment	NR	They compared tertiles or quintiles: TG < 83.4 mg/dL (< 0.94), 84.2-117.8 mg/L (0.95-1.33), and > 118.7 mg/dL (1.34 mmol/L)

Carlson, 1988[18]	consecutive sample (all patients presenting with HTG)	chart review, ascertained the same way in cases and controls, done without knowledge of patients' TG level	NR	13.4%	3 groups according to TG levels. Low = TG < 132.9 mg/dL (1.5 mmol/L), intermediate = TG 132.9-177.2 mg/dL (1.5-2.0 mmol/L), high = TG > 177.2 (2.0 mmol/L).

Chan, 2005[19]	not random; consecutive patients with type 2 DM, not HPTG	chart review, death registry	Adjusted for sex and age. stepwise linear regression with BMI, WC, HbA1c, FPG and HOMA as independent variables and lipid profile as dependent variable	0	Unclear

Chester, 1995[20]	consecutive sample (all men presenting with HTG and are awaiting routine angioplasty)	chart review, done without knowledge of patients' TG level	The potential predictor variables-that is, risk factors assessed at baseline angiography, for adverse events were analyzed using the multiple logistic regression models.	2	TG value in the blood was used as a continuous number (variable). Here OR expresses increased risk per unit of serum TG level: mmol/L

Czernichow, 2007[21]	consecutive sample	self report, chart review, ICD or billing codes, ascertained differently: self report in all patients, however if a CVD event was reported -- chart review and ICD billing codes were reviewed for those individuals only	Age	NR	Age-adjusted relative risk correlate to one standard deviation increase in TG levels

Drexel, 2005[22]	consecutive sample	follow up investigation after 2.3 years, Time and causes of death were obtained from national surveys, hospital records	age, sex, and use of lipid-lowering medication	0	Unclear

Eberly, 2003[23]	not random; likely consecutive sample: 2863 men with both nonfasting and fasting TG levels measured at screens 1 and 2	self report, chart review, ICD or billing codes, death certificates	age, lipids subfractions, glucose level, BP, cigarettes smoked per day, alcohol use, BMI and race	0	TG value in the blood was used as a continuous number (variable). Here OR expresses increased risk per unit of serum TG level: mg/dL

Egger, 1999[24]	not random; likely consecutive sample: Participants of the Caerphilly Heart Disease Study	self report, chart review, ICD or billing codes	age, all three lipid factors, laboratory error and within person variation, blood glucose and diastolic BP, BMI, smoking and markers for pre-existent disease	12.5	TG value in the blood was used as a continuous number (variable). Here OR expresses increased risk per unit of serum TG level: mmol/L

Ellingsen, 2003[25]	not random; likely consecutive sample: 1232 healthy men with elevated cholesterol or coronary risk score included in the study from a pool of 16202 screened men	chart review, ICD or billing codes, ascertained the same way in cases and controls	adjusted for age, BMI, cigarette smoking, total cholesterol, triacylglycerol, glucose, BP, dietary score, alcohol intake, and activity level	0	TG value in the blood was used as a continuous number (variable). Here OR expresses increased risk per unit of serum TG level: high TG > or = 178.1 mg/dL (2.01 mmol/L)

Gaziano, 1997[26]	not random, likely consecutive sample: Men/women < 76 yrs. age with no prior history of CAD discharged from one of 6 Boston area hospitals with the diagnosis of confirmed MI	chart review, medical exam/lab analysis, ascertained differently: cases were interviewed 8 weeks after MI	Adjusted for age, sex, history of HTN, history of DM, body mass index, type A personality, family history of previous MI, alcohol consumption, physical activity, smoking, caloric intake	12	they compared quintiles, highest compared to lowest

Goldberg, 2009[27]	consecutive sample (all patients presenting with HTG)	chart review, telephone calls, ascertained the same way in cases and controls	A time-to-event regression model was performed to establish the role of baseline lipid subfractions, other metabolic risk factors, lifestyle variables, and demographic characteristics in relation to the development of CAD.	3.8	high triglyceride level > = 248.1 mg/dL (2.8 mmol/L)

Habib, 2006[28]	Data from the United States Renal Data System database collected during the prospective Dialysis Morbidity and Mortality Study Wave 2 study	chart review	age, gender, race, weight, height, primary cause of ESRD, hemoglobin, serum albumin, serum calcium phosphate product, serum bicarbonate, residual kidney creatinine clearance, PD parameters (dialysate effluent volume, dialysis creatinine clearance, D/P creatinine ratio after a 4 h dwell), use of lipid-modifying medications and comorbidity characteristics	0	TG value in the blood was used as a continuous number (variable). Here OR expresses increased risk per unit of serum TG level: HR is using a reference of TG levels 200-300 mg/dl (2.2-3.4 mmol/L)

Haim, 1999[29]	not random; likely consecutive sample	chart review, ICD or billing codes	age, previous MI, DM, NYHA class, HTN, LDL cholesterol, glucose, chronic obstructive pulmonary disease, peripheral vascular disease, stroke, angina pectoris, smoking, and lipids	0.37	TG value in the blood was used as a continuous number (variable). Here OR expresses increased risk per unit of serum TG level: mg/dL

Hoogeveen, 2001[30]	Random sample	chart review, clinical exam and investigations, ascertained the same way in cases and controls	Logistic regression applied but no specific adjustments are mentioned	12	TG value in the blood was used as a continuous number (variable). Here OR expresses increased risk per unit of serum TG level: 10 mg/dL (0.11 mmol/L)

Jonsdottir, 2002[31]	not random; likely consecutive: subjects of the Reykjavik Study	self report, chart review, ICD or billing codes	age, high-density lipoprotein cholesterol, total/low-density lipoprotein cholesterol, smoking, body mass index and BP	0.6	TG value in the blood was used as a continuous number (variable). Here OR expresses increased risk per unit of serum TG level: mmol/L

Lamarche, 1995[32]	random sample	chart review, Examination/EKG/death certificate	Adjusted for age, systolic BP, DM, alcohol consumption, and tobacco use	27	TG value in the blood was used as a continuous number (variable). Here OR expresses increased risk per unit of serum TG level: TG > 203.8 mg/dL (2.3 mmol/L)

Lloret Linares, 2008[33]	not random and not consecutive: Patients referred by their general practitioner or general hospital for very high TG levels to Endocrinology Dept. between 2000 and 2005	self report, chart review	Adjusted for age at hospitalization	NR	TG: lowest 95.1-180 mg/dL (1.1-2.0 mmol/L) vs. highest 360-1505 gm/dL (4.1-17 mmol/L).

Lu, 2003[34]	not random; likely consecutive: cohort chosen from the strong heart study to include only DM, no baseline CVD	through death certificates and tribal and Indian Health Service hospital records and by direct contact of study personnel with the study participants and their families	Adjusted for age, BMI, smoking status, study center, systolic BP, HbA1c, fibrinogen, insulin, and ratio of albumin to creatinine	0	They compared tertiles or quintiles: TG: lower < 111; 111- 175; higher > 175 mg/dL (lower < 1.2; 1.2-2.0; higher > 2.0 mmol/L)

Malone, 2009[35]	Not random; likely consecutive: Retrospective data from 3 integrated health-care systems that systematically collect and store detailed patient-level data.	Chart review, ICD or billing codes	Adjusted for age, sex, smoking status and site	N/A	lower/normal TG - 80.0 mg/dl (0.9 mmol/L); higher TG - TG = 217.4 mg/dl (2.4 mmol/L)

Mazza, 2005[36]	random sample	chart review, ICD or billing codes, through the Register Office, general practitioners	Gender, age, DM, obesity, lipids subfractions, serum uric acid, BP, smoking, alcohol and proteinuria	0	They compared tertiles or quintiles: TG: First (low) < 97.5 mg/dL (1.01 mmol/L); Fifth (high) > = 156.8 mg/dL (1.77 mmol/L)

Mora, 2008[37]	Random sample enrolled in the Women's Health Study	Follow-up questionnaires every 6- 12 months	Adjusted for age, randomized treatment assignment, smoking status, menopausal status, postmenopausal hormone use, BP, DM, and BMI	NR	They compared tertiles or quintiles: TG: First (low) < 89.5 mg/dL (1.01 mmol/L); Fifth (high) > = 180.7 mg/dL (2.04 mmol/L)

Noda, 2010[38]	not random and not consecutive: death related to a MI defined a case, then 2 controls were selected randomly matched by age	Death registration from 1997-2000, done without knowledge of patients' TG level, ascertained the same way in cases and controls	Adjusted for age and 6 risk factors for MI	NR	TG value in the blood was used as a continuous number (variable). Here OR expresses increased risk per unit of serum TG level: High TG > = 150 mg/dL (1.7 mmoml/L)

Rubins, 1999[39]	not random and not consecutive: to obtain population with appropriate lipid levels, a multi stage screening method that included two lipid profiles obtained one week apart	chart review, clinical and radiologic data, ascertained the same way in cases and controls	Adjustment for baseline variables in the Cox models had a trivial effect on the estimates of the hazard ratios	2.3%	Two groups: TG < 150 mg/dl (1.7 mmol/L) and TG > 150 mg/dl (1.7 mmol/L)

Samuelsson, 1994[40]	random sample	chart review	traditional risk factors, end-organ damage status	NR	TG value in the blood was used as a continuous number (variable). Here OR expresses increased risk per unit of serum TG level: RR reported for every 88.6 mg/dL (1 mmol/L) increase in TG level

Schupf, 2005[41]	random sample	self report, chart review, interviewing relatives	Adjusted for age, sex, ethnicity, and level of education, for BMI or APOE; a history of HTN, DM, heart disease, stroke, or cancer; or current smoking	0	They compared tertiles or quintiles: Lowest - < = 98.9 mg/dl (1.1 mmol/L), highest - > 191.2 mg/dl (2.1 mmol/L); RR compared the lowest quartile to the highest quartile.

Sprecher, 2000[42]	not random; likely consecutive: diabetic patients undergoing primary isolated CABG between 1982 and 1992 at Cleveland Clinic	chart review, clinical exam, labs and CVIR (Cardiovascular Information Registry)	age, sex, left ventricular function, coronary anatomy, history of HTN, BMI, and total cholesterol	NR	highest quartile compared to lower three quartiles (normal)

Tanko, 2005[43]	not random and not consecutive: recruited via a questionnaire surveys	self report, chart review: Central Registry of the Danish Ministry of Health	Adjusted for age, smoking, and LDL-C), waist circumference	NR	TG value in the blood was used as a continuous number (variable). Here OR expresses increased risk per unit of serum TG level: presented as 2 cutoffs - > 128.5 mg/dL (1.45 mmol/L) - > 149.7 mg/dL (1.69 mmol/L)

Tsai, 2008[44]	not random; likely consecutive: civil servants and teachers who took the annual physical examination at the Taipei Outpatient Center	chart review, annual exam, national death files	Adjusted for age, gender, fasting glucose, BP, BMI, smoking	NR	They compared tertiles or quintiles: TG normal < 150 mg/dL (1.7 mmol/L, abnormal 150 mg/dL (1.7 mmol/L)-199 mg/dL (2.2 mmol/L), and high abnormal > 200 mg/dL (2.25 mmol/L)

Upmeier, 2009[45]	not random and not consecutive: mailed invitation to participate to all residents of Turku born in 1920	Self report, chart review, ICD or billing codes	Adjusted for gender, body mass index, smoking and any history of angina pectoris, stroke, DM, and HTN	NR	They compared TG level quartiles, highest to lowest

Valdivielso, 2009[46]	not random; likely consecutive	Chart review and self report	age, sex, smoking, HTN, DM, and lipids fractions	NR	Categorized as normal when TG was < 150 mg/dL (< 1.69 mmol/L); the remainder were considered to be HTG

Wier, 2003[47]	not random; likely consecutive	chart review, done without knowledge of patients' TG level	age, time of resolution of symptoms, smoking, BP, presence of atrial fibrillation and hyperglycemia	0	They compared tertiles or quintiles: TG, mmol/l: < = 0.9; 1.0-1.3; 1.4-1.8; > = 1.9. Mg/dL: < = 79.7; 88.6-115.2; 124.0-159.5; > = 168.3

UC/NR: unclear, not reported; TG: Triglycerides; HTG, hypertriglyceridemia; MI, myocardial infarction; DM, diabetes, BP, blood pressure, HTN, hypertension

* It was unclear in most studies if enrolled patients did not have the outcomes pre-existent at baseline. In most studies, it was also unclear if patients were treated with drugs that can affect TG level (both of these elements lower the observed strength of association)

Triglycerides Conversion from mg/dL to mmol/L: multiply by (x) 0.01129; from mmol/L to mg/dL: multiply by (x) 88.6

### Meta-analysis

**Figure 2:**
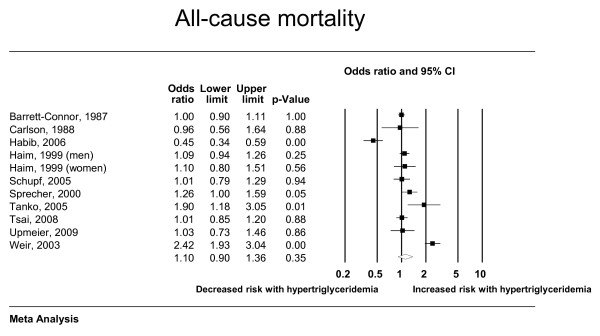
**Random effects meta-analysis (all-cause mortality)**.

**Figure 3:**
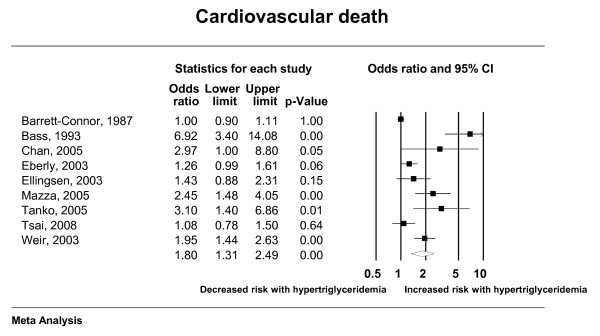
**Random effects meta-analysis (cardiovascular death)**.

**Figure 4:**
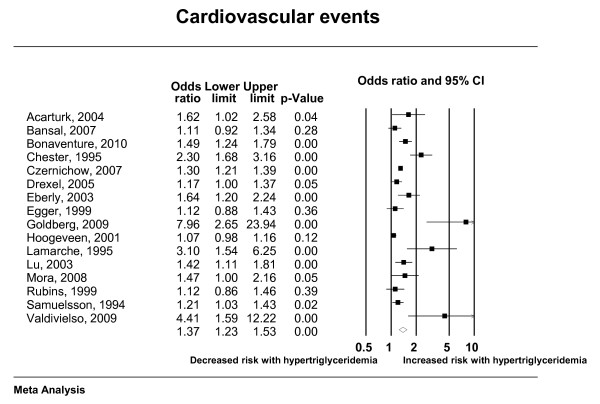
**Random effects meta-analysis (cardiac events)**.

**Figure 5:**
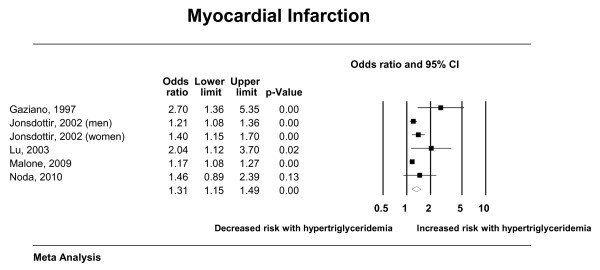
**Random effects meta-analysis (myocardial infarction)**.

The total number of included studies was 35 enrolling 927,218 patients who suffered 132,460 deaths and 72,654 cardiac events; respectively. Hypertriglyceridemia was significantly associated with cardiovascular death, cardiovascular events, myocardial infarction, and pancreatitis; with odds ratios (95% confidence interval) of 1.80 (1.31-2.49), 1.37 (1.23-1.53), 1.31 (1.15-1.49) and 3.96 (1.27-12.34); respectively. There was nonsignificant association with all-cause mortality (OR: 1.10; 95% CI: 0.90-1.36). Forest plots depicting the results of random effects meta-analysis are presented in Figures 2, 3, 4 and 5.

It is worth noting that the association with acute pancreatitis was estimated by only one eligible study that included 129 patients with severe hypertriglyceridemia (119 with type IV phenotypes and 10 with type V phenotypes according to Fredrickson's classification) of whom 26 suffered acute pancreatitis [33]. In this study, subjects in the third tertile of TG had a 4.0-fold increased risk (95% confidence interval, 1.3-12.3) compared with the first tertile and those diagnosed with dyslipidemia at a younger age also had increased risk.

All analyses were associated with important heterogeneity (*I^2 ^*> 50%) that our planned subgroup analyses could only partially explain (Table 3). The association of hypertriglyceridemia with mortality and cardiovascular mortality seemed to be stronger in women. These findings are consistent with a previous meta-analysis published in 1996. Hokanson and Austin estimated adjusted relative risks for incident cardiovascular events of 1.14 (95% Cl 1.05-1.28) in men and 1.37 (95% Cl 1.13-1.66) in women. The association with cardiovascular events was somewhat stronger in patients with diabetes although this effect was not statistically significant. Hence, there were no other significant subgroup interactions to explain heterogeneity (based on the level of adjustment for lipids subfractions, sex or the presence of diabetes).

**Table 3:** Subgroup analysis

Subgroup	**No**. studies	OR	LL	UL	P-effect Size	P- interaction
**Mortality**						

Men	3	1.03	0.95	1.12	0.49	0.04

Women	3	1.55	1.05	2.27	0.03	

adequate adjustment	9	1.09	0.83	1.43	0.55	0.54

inadequate adjustment	3	1.22	0.94	1.59	0.14	

General population	10	1.09	0.87	1.37	0.46	0.49

Diabetes	2	1.37	0.75	2.50	0.31	

**Cardiovascular death**						

Men	3	1.14	0.92	1.40	0.23	0.00

Women	2	4.73	2.15	10.37	0.00	

adequate adjustment	5	1.88	1.12	3.15	0.02	0.84

inadequate adjustment	4	1.76	1.18	2.62	0.01	

General population	8	1.75	1.26	2.43	0.00	0.36

Diabetes	1	2.97	1.00	8.80	0.05	

**Cardiovascular events**						

Men	6	1.29	1.13	1.47	0.00	0.67

Women	2	1.21	0.94	1.57	0.14	

adequate adjustment	12	1.39	1.23	1.58	0.00	0.91

inadequate adjustment	4	1.37	1.01	1.84	0.04	

General population	15	1.37	1.22	1.54	0.00	0.81

Diabetes	1	1.42	1.11	1.81	0.00	

**Myocardial infarction**						

Men	2	1.22	1.09	1.37	0.00	0.24

Women	1	1.40	1.15	1.70	0.00	

adequate adjustment	3	1.72	0.98	3.01	0.06	0.29

inadequate adjustment	3	1.26	1.15	1.39	0.00	

General population	5	1.27	1.13	1.44	0.00	0.13

Diabetes	1	2.04	1.12	3.70	0.02	

*Only feasible analyses are shown

There was no evidence of publication bias (P value for Eggers test > 0.05 for all outcomes) although these analyses were underpowered to detect this problem and the presence of heterogeneity further limits the ability to detect publication bias.

## Discussion

We conducted a systematic review and meta-analysis and documented an association between fasting hypertriglyceridemia and the risk of several cardiovascular adverse events and with pancreatitis.

### Limitations, strengths and comparison with other reports

The main limitation of association studies is the observational nature of the existing evidence. Therefore, confounders (particularly, baseline risk of patients for developing cardiovascular disease and the effect of other lipid subfractions abnormalities) threaten the validity of results. In meta-analyses of observational studies, the ability to adjust for confounding is limited by the level of adjustment conducted in the original studies. We attempted to evaluate confounding by conducting subgroup analysis; however, this analysis was underpowered. Other limitations pertain to heterogeneity of the meta-analytic estimates, publication bias (which remains likely in the context of observational studies that do not require prospective registration) and reporting bias (which is also likely considering that several studies met the eligibility criteria for this review but did not report the outcomes of interest) [48]. It was unclear in most studies if enrolled patients did not have some of the outcomes pre-existent at baseline and it was also unclear if patients were treated with drugs that can affect TG level (both of these elements lower the confidence in the observed associations). We only found one controlled study that evaluated the association with acute pancreatitis.

The overall confidence in the estimated magnitude of associations is low [10]considering the described methodological limitations in evaluating the association with cardiovascular events; and imprecision (small number of events) in evaluating the association with pancreatitis.

The strengths of this study stems from the comprehensive literature search that spans across multiple databases and duplicate appraisal and study selection. Our results are consistent with previous evidence synthesis reports about the association of hypertriglyceridemia with cardiovascular events. We estimated increased odds by 37% (odds ratio of 1.37). Hokanson and Austin [3] estimated adjusted relative risks of 1.14 (95% Cl 1.05-1.28) in men and 1.37 (95% Cl 1.13-1.66) in women. Sarwar et al. [2] reported odds ratio of 1.73 in prospective cohort studies published prior to 2006. A systematic review by Labreuche et al. [49] demonstrated that baseline triglyceride levels in randomized trials is associated with increased stroke risk (adjusted RR, 1.05 per 10-mg/dL (0.1 mmol/L) increase; 95% CI, 1.03-1.07). To our knowledge, this is the first systematic review that sought to identify controlled studies evaluating the association with pancreatitis.

### Implications

The associations demonstrated between hypertriglyceridemia and cardiovascular risk should not necessarily translate into a recommendation for treatment. It is plausible that the benefits of lowering triglycerides do not merely depend on how much the level is lowered, but rather on how it is lowered (i.e., lifestyle interventions vs. pharmacological therapy). Therefore, randomized trials of the different approaches with patient-important outcomes [50] used as primary endpoints are needed for making policy and clinical decisions.

Several systematic reviews and meta-analyses [49,51-54] have summarized the evidence from randomized trials of fibrate therapy and demonstrated that fibrate therapy reduced the risk of vascular events (RR 0.75, 95% CI 0.65 to 0.86) in patients with high triglyceride levels or atherogenic dyslipidemia (low HDL cholesterol combined with high triglyceride level) although all-cause mortality and non cardiovascular mortality were both significantly increased in clofibrate trials. Meta-analyses [55,56] of niacin therapy demonstrate significant reduction in the risk of major coronary events (25% reduction in relative odds; 95% CI 13, 35), stroke (26%; 95% CI 8, 41) and any cardiovascular events (27%; 95% CI 15, 37). However, contemporary trials in the statin era have failed to substantiate these findings with fenofibrate among patients with diabetes [57] and with niacin in high risk patients [58]. Also, to our knowledge, there are no trials assessing the value of triglyceride lowering to reduce the risk of pancreatitis. Thus, lifestyle changes should remain the mainstay of therapy. Treatment of the underlying metabolic disorder (e.g., insulin resistance) should also be an essential and first step in the management plan of hypertriglyceridemia.

## Conclusions

The current evidence suggests that hypertriglyceridemia is associated with increased risk of cardiovascular death, MI, cardiovascular events, and acute pancreatitis. The strength of inference is limited by the unexplained inconsistency of results and high risk of confounding and publication bias.

## Competing interests

The authors declare that they have no competing interests.

## Authors' contributions

MHM, VMM, LB and PJE conceived and designed the study and acquired funding. HM, AH, FCY, SD, SG, IB, ML and TE collected data. MHM, VMM and AH conducted analysis. MHM, VMM and LB drafted the manuscript. All authors provided critical revisions to the manuscript and made substantive intellectual contributions to the study. All authors read and approved the final manuscript.

## Pre-publication history

The pre-publication history for this paper can be accessed here:

http://www.biomedcentral.com/1472-6823/12/2/prepub

## Supplementary Material

Additional file 1 **Search Strategy**. Click here for file
