# Supplementary material for: The association of hypertriglyceridemia with cardiovascular events and pancreatitis: a systematic review and meta-analysis
Source: BMC Endocr Disord. 2012 Mar 31;12:2. doi: 10.1186/1472-6823-12-2 (PMC3342117; doi:10.1186/1472-6823-12-2)
Supplement: Additional file 1 — Search Strategy. [file 1472-6823-12-2-S1.DOC]

**Search Strategy:**

Triglyceride* adj3 (level* or blood or concentrat* or elevat* or increase*)
Proportional hazards model?

Ovid MEDLINE(R) 1950 to July Week 4 2010 # Searches Results Search Type 1
exp Triglycerides/an, bl, du [Analysis, Blood, Diagnostic Use] 39717 Advanced 2
exp Hypertriglyceridemia/bl, di, ep, eh, mo, pa [Blood, Diagnosis, Epidemiology, Ethnology, Mortality, Pathology] 2151 Advanced 3

(triglycerid* adj3 (level* or blood* or serum or increas* or elevat* or concentrat* or high)).mp. [mp=title, original title, abstract, name of substance word, subject heading word, unique identifier] 29534 Advanced 4

1 or 2 or 3 57117 Advanced 5
exp Cardiovascular Diseases/ 1584926 Advanced 6
exp Cerebrovascular Disorders/ 224932 Advanced 7
exp Cardiac Surgical Procedures/ 137672 Advanced 8
exp Stents/ 38286 Advanced 9
exp arterial occlusive diseases/ or exp "embolism and thrombosis"/ or exp hypertension/ or exp myocardial ischemia/ 710632 Advanced 10

4 and (5 or 6 or 7 or 8 or 9) 16809 Advanced 11
10 and (cohort* or population* or observation* or placebo*).mp. [mp=title, original title, abstract, name of substance word, subject heading word, unique identifier] 4869 Advanced 12

4 and exp pancreatitis/ 420 Advanced 13
12 and (cohort* or population* or observation* or placebo*).mp. [mp=title, original title, abstract, name of substance word, subject heading word, unique identifier] 35 Advanced 14

11 and randomized controlled trial.pt. 654 Advanced 15
exp *Triglycerides/an, bl, du or exp *Hypertriglyceridemia/bl, di, ep, eh, mo, pa or (triglycerid* adj3 (level* or blood* or serum or increas* or elevat* or concentrat* or high)).tw. 33649 Advanced 16

11 and 15 3408 Advanced 17
16 and (mo.fs. or mortal*.mp. or morbid*.mp. or death*.mp. or risk*.mp.) [mp=title, original title, abstract, name of substance word, subject heading word, unique identifier] 2723 Advanced 18

"all cause".mp. and 16 [mp=title, original title, abstract, name of substance word, subject heading word, unique identifier] 44 Advanced 19

13 or 14 or 17 or 18 3151 Advanced 20
limit 19 to humans 3134 Advanced 21
exp Cardiovascular Diseases/ep, co, mo or exp Cerebrovascular Disorders/ep, co, mo or exp Cardiac Surgical Procedures/ep, co, mo or exp Stents/ep, co, mo or (exp arterial occlusive diseases/ep, co, mo or exp "embolism and thrombosis"/ep, co, mo or exp hypertension/ep, co, mo or exp myocardial ischemia/ep, co, mo) 416836 Advanced 22

20 and 21 1545 Advanced 23
20 and ((death* or mortality or morbid*).mp. or mo.fs. or event*1.mp.) [mp=title, original title, abstract, name of substance word, subject heading word, unique identifier] 951 Advanced 24

22 or 23 1913 Advanced 25
20 and exp cohort studies/ 868 Advanced 26
24 or 25 2140 Advanced

EMBASE 1988 to 2010 Week 31 # Searches Results Search Type 1
hypertriglyceridemia/di, ep, et [Diagnosis, Epidemiology, Etiology] 1789 Advanced 2
triacylglycerol blood level/ 24606 Advanced 3
(triglyceride* adj3 (level* or blood or concentrat* or elevat* or increas* or high or range)).mp. [mp=title, abstract, subject headings, heading word, drug trade name, original title, device manufacturer, drug manufacturer] 25566 Advanced 4

or/1-3 41730 Advanced 5
cardiovascular risk/ 68625 Advanced 6
exp coronary artery surgery/ or exp bypass surgery/ or exp heart surgery/ 218109 Advanced 7
exp cardiovascular disease/ 1617998 Advanced 8
exp STROKE/ 80374 Advanced 9
5 or 6 or 7 or 8 1702399 Advanced 10
4 and 9 20212 Advanced 11
10 and (cohort*.mp. or major clinical study/ or observational*.mp. or population*.mp.) [mp=title, abstract, subject headings, heading word, drug trade name, original title, device manufacturer, drug manufacturer] 9628 Advanced 12

*hypertriglyceridemia/di, ep, et or *triacylglycerol blood level/ or (triglyceride* adj3 (level* or blood or concentrat* or elevat* or increas* or high or range)).tw. 26482 Advanced 13

11 and 12 5886 Advanced 14
13 and pancreatitis*.mp. [mp=title, abstract, subject headings, heading word, drug trade name, original title, device manufacturer, drug manufacturer] 27 Advanced 15

13 and (ep.fs. or mortality.mp. or death*.mp. or event*.mp. or fatal*.mp. or nonfatal*.mp.) [mp=title, abstract, subject headings, heading word, drug trade name, original title, device manufacturer, drug manufacturer] 2429 Advanced 16

13 and placebo*.mp. [mp=title, abstract, subject headings, heading word, drug trade name, original title, device manufacturer, drug manufacturer] 359 Advanced 17

14 or 15 or 16 2607 Advanced 18
17 not case report/ 2605 Advanced 19
(*cardiovascular risk/ or (exp *coronary artery surgery/ or exp *bypass surgery/ or exp *heart surgery/) or exp *cardiovascular disease/ or exp *STROKE/) and 12 8550 Advanced 20

exp clinical trial/ or exp major clinical study/ or exp prospective study/ or exp retrospective study/ 1981247 Advanced 21

19 and 20 4088 Advanced 22
17 and 21 1533 Advanced 23
18 and (cohort*.mp. or major clinical study/ or observational*.mp. or population*.mp.) [mp=title, abstract, subject headings, heading word, drug trade name, original title, device manufacturer, drug manufacturer] 2605 Advanced 24

22 or 23 2605 Advanced
25 limit 24 to human 2540  Advanced Display
26 21 and (meta-analysis/ or systematic review/) 64  Advanced Display
27 25 or 26 2586  Advanced
SCOPUS
Your query: (TITLE-ABS-KEY(((tg OR triglyceride*) AND (fasting OR nonfasting OR blood OR serum OR level* OR concentrat* OR increas* OR elevat* OR high*))) AND TITLE-ABS-KEY((cardiovasc* OR heart OR coronary OR myocard* OR stroke OR cerebrovasc* OR atheroscler* OR pancreatitis OR hypertens*) AND (death* OR mortal* OR event OR events OR dying OR incidence OR "relative risk" OR "risk assessment")) AND TITLE-ABS-KEY((cohort* OR population OR placebo* OR arm))) AND NOT PMID(1* OR 2* OR 3* OR 4* OR 5* OR 6* OR 7* OR 8* OR 9*) AND NOT rats
